# Supplementary material for: Characterization of spatio-temporal dynamics of the constrained network of the filamentous fungus Podospora anserina using a geomatics-based approach
Source: PLoS One. 2024 Feb 6;19(2):e0297816. doi: 10.1371/journal.pone.0297816 (PMC10846696; doi:10.1371/journal.pone.0297816)
Supplement: S1 Table — The exponential fit is Xi(t)=Xi02(t+t0,i)/τi, where X stands respectively for A, N or L and i for experiment i = 1, 2 or 3. t is time and t0,i is the temporal offset. τi is the characteristic growth time. The uncertainty represents one standard deviation. (PDF) [file pone.0297816.s001.pdf]

S1 Table. Parameter values of the exponential fit for the three replicates in conditions 0 to 5. The exponential fit is $X_i(t) = X_i^0\, 2^{(t+t_{0,i})/\tau_i}$, where X stands respectively for A, N or L and i for experiment $i = 1$, 2 or 3. t is time and $t_{0,i}$ is the temporal offset. τ_i is the characteristic growth time. The uncertainty represents one standard deviation.

	Exp	t_0 [h]	A_0	τ_A [h]	χ^2_A	N_0	τ_N [h]	χ^2_N	L_0 [mm]	τ_L [h]	χ^2_L
Condition 0	1	0	$2,03 \pm 0,46$	$2,09 \pm 0,12$	$3,2$	$1,07 \pm 0,35$	$1,68 \pm 0,08$	$7,4$	$0,84 \pm 0,30$	$2,03 \pm 0,13$	$5,7$
	2	0,9	$3,01 \pm 0,54$	$2,15 \pm 0,10$	$8,2$	$1,60 \pm 0,50$	$1,63 \pm 0,06$	$7,7$	$0,91 \pm 0,31$	$1,95 \pm 0,11$	$3,9$
	3	0	$2,38 \pm 0,48$	$2,03 \pm 0,11$	$2,8$	$1,21 \pm 0,37$	$1,57 \pm 0,07$	$6,3$	$0,88 \pm 0,31$	$1,92 \pm 0,12$	$5,5$
Condition 1	1	0	$2,46 \pm 0,52$	$2,67 \pm 0,15$	$1,1$	$1,44 \pm 0,45$	$2,13 \pm 0,10$	$3,8$	$1,09 \pm 0,38$	$2,67 \pm 0,18$	$4,2$
	2	0	$2,71 \pm 0,52$	$3,07 \pm 0,17$	$5,4$	$1,68 \pm 0,52$	$2,50 \pm 0,13$	$3,9$	$1,05 \pm 0,36$	$2,91 \pm 0,20$	$9,4$
	3	1,5	$2,70 \pm 0,51$	$3,04 \pm 0,15$	$7,6$	$1,89 \pm 0,56$	$2,55 \pm 0,11$	$4,8$	$0,83 \pm 0,29$	$2,80 \pm 0,17$	$2,9$
Condition 2	1	1,8	$3,01 \pm 0,53$	$3,00 \pm 0,15$	$2,6$	$1,78 \pm 0,48$	$2,28 \pm 0,09$	$10,0$	$0,75 \pm 0,26$	$2,75 \pm 0,17$	$8,2$
	2	0	$1,91 \pm 0,48$	$2,89 \pm 0,24$	$4,3$	$1,68 \pm 0,57$	$2,13 \pm 0,16$	$1,9$	$0,45 \pm 0,22$	$2,47 \pm 0,26$	$3,7$
	3	0	$2,10 \pm 0,38$	$3,06 \pm 0,13$	$2,7$	$1,34 \pm 0,36$	$2,32 \pm 0,08$	$5,0$	$0,66 \pm 0,22$	$2,78 \pm 0,15$	$1,1$
Condition 3	1	2,2	$1,65 \pm 0,57$	$2,66 \pm 0,30$	$2,9$	$0,61 \pm 0,27$	$1,85 \pm 0,15$	$9,4$	$0,29 \pm 0,19$	$2,61 \pm 0,42$	$3,2$
	2	3,7	$2,78 \pm 0,61$	$3,74 \pm 0,28$	$4,4$	$1,20 \pm 0,38$	$2,65 \pm 0,13$	$1,6$	$0,39 \pm 0,20$	$3,28 \pm 0,38$	$6,8$
	3	2,2	$2,81 \pm 0,74$	$3,61 \pm 0,35$	$4,1$	$1,08 \pm 0,37$	$2,29 \pm 0,14$	$5,7$	$0,38 \pm 0,21$	$3,13 \pm 0,42$	$1,3$
Condition 4	1	0	$2,04 \pm 0,45$	$3,50 \pm 0,26$	$3,3$	$0,80 \pm 0,27$	$2,31 \pm 0,13$	$2,9$	$0,43 \pm 0,19$	$2,91 \pm 0,27$	$9,3$
	2	0,9	$2,61 \pm 0,66$	$3,88 \pm 0,39$	$7,2$	$1,83 \pm 0,61$	$2,73 \pm 0,19$	$6,4$	$0,79 \pm 0,35$	$3,51 \pm 0,42$	$6,0$
	3	0,3	$2,58 \pm 0,52$	$3,72 \pm 0,24$	$6,8$	$1,72 \pm 0,51$	$2,79 \pm 0,14$	$2,7$	$0,86 \pm 0,31$	$3,35 \pm 0,26$	$1,9$
Condition 5	1	1,2	$2,52 \pm 0,61$	$1,92 \pm 0,13$	$2,8$	$1,20 \pm 0,40$	$1,45 \pm 0,07$	$4,1$	$0,70 \pm 0,30$	$1,91 \pm 0,17$	$3,6$
	2	2,4	$2,68 \pm 0,68$	$2,12 \pm 0,15$	$7,4$	$1,68 \pm 0,56$	$1,68 \pm 0,09$	$2,7$	$0,90 \pm 0,37$	$2,17 \pm 0,19$	$1,8$
	3	1,6	$3,01 \pm 0,40$	$2,00 \pm 0,08$	$2,4$	$2,64 \pm 0,54$	$1,70 \pm 0,06$	$4,5$	$1,01 \pm 0,30$	$2,06 \pm 0,14$	$2,8$
